# Innovative prognostic modeling in ESCC: leveraging scRNA-seq and bulk-RNA for dendritic cell heterogeneity analysis

**DOI:** 10.3389/fimmu.2024.1352454

**Published:** 2024-03-06

**Authors:** Mengnan Shi, Han Zhang, Linnan Ma, Xiaoting Wang, Daqiang Sun, Zhijie Feng

**Affiliations:** ^1^ Department of Gastroenterology, The Second Hospital of Hebei Medical University, Shijiazhuang, Hebei, China; ^2^ Hebei Key Laboratory of Gastroenterology, Hebei Institute of Gastroenterology, Shijiazhuang, Hebei, China; ^3^ Hebei Clinical Research Center for Digestive Diseases, Hebei Institute of Gastroenterology, Shijiazhuang, Hebei, China; ^4^ Clinical School of Thoracic, Tianjin Medical University, Tianjin, China; ^5^ Tianjin Chest Hospital, Tianjin University, Tianjin, China

**Keywords:** ESCC, TME, cDC, pDC, TDC, immunotherapy

## Abstract

**Background:**

Globally, esophageal squamous cell carcinoma (ESCC) stands out as a common cancer type, characterized by its notably high rates of occurrence and mortality. Recent advancements in treatment methods, including immunotherapy, have shown promise, yet the prognosis remains poor. In the context of tumor development and treatment outcomes, the tumor microenvironment (TME), especially the function of dendritic cells (DCs), is significantly influential. Our study aims to delve deeper into the heterogeneity of DCs in ESCC using single-cell RNA sequencing (scRNA-seq) and bulk RNA analysis.

**Methods:**

In the scRNA-seq analysis, we utilized the SCP package for result visualization and functional enrichment analysis of cell subpopulations. CellChat was employed to identify potential oncogenic mechanisms in DCs, while Monocle 2 traced the evolutionary trajectory of the three DC subtypes. CopyKAT assessed the benign or malignant nature of cells, and SCENIC conducted transcription factor regulatory network analysis, offering a preliminary exploration of DC heterogeneity. In Bulk-RNA analysis, we constructed a prognostic model for ESCC prognosis and immunotherapy response, based on DC marker genes. This model was validated through quantitative PCR (qPCR) and immunohistochemistry (IHC), confirming the gene expression levels.

**Results:**

In this study, through intercellular communication analysis, we identified GALECTIN and MHC-I signaling pathways as potential oncogenic mechanisms within dendritic cells. We categorized DCs into three subtypes: plasmacytoid (pDC), conventional (cDC), and tolerogenic (tDC). Our findings revealed that pDCs exhibited an increased proportion of cells in the G2/M and S phases, indicating enhanced cellular activity. Pseudotime trajectory analysis demonstrated that cDCs were in early stages of differentiation, whereas tDCs were in more advanced stages, with pDCs distributed across both early and late differentiation phases. Prognostic analysis highlighted a significant correlation between pDCs and tDCs with the prognosis of ESCC (P< 0.05), while no significant correlation was observed between cDCs and ESCC prognosis (P = 0.31). The analysis of cell malignancy showed the lowest proportion of malignant cells in cDCs (17%), followed by pDCs (29%), and the highest in tDCs (48%), with these results being statistically significant (P< 0.05). We developed a robust ESCC prognostic model based on marker genes of pDCs and tDCs in the GSE53624 cohort (n = 119), which was validated in the TCGA-ESCC cohort (n = 139) and the IMvigor210 immunotherapy cohort (n = 298) (P< 0.05). Additionally, we supplemented the study with a novel nomogram that integrates clinical features and risk assessments. Finally, the expression levels of genes involved in the model were validated using qPCR (n = 8) and IHC (n = 16), thereby confirming the accuracy of our analysis.

**Conclusion:**

This study enhances the understanding of dendritic cell heterogeneity in ESCC and its impact on patient prognosis. The insights gained from scRNA-seq and Bulk-RNA analysis contribute to the development of novel biomarkers and therapeutic targets. Our prognostic models based on DC-related gene signatures hold promise for improving ESCC patient stratification and guiding treatment decisions.

## 1 Introduction

ESCC holds a notable position in global cancer statistics, being the seventh most common in terms of new cases and the sixth leading cause of cancer-related deaths (1). In the year 2020, the worldwide incidence of esophageal cancer was around 604,000, leading to approximately 544,076 fatalities. Notably, more than half of these instances were reported in China (2). Approximately 90% of esophageal cancers are of the squamous cell carcinoma type. Despite advancements in treatment methods, the prognosis for ESCC remains concerning, with a relatively low overall five-year survival rate. Currently, the primary treatment modalities for ESCC include surgery, chemotherapy, radiotherapy, and limited targeted therapy (3). However, these treatments offer only limited survival benefits. In recent years, the advent of cancer immunotherapy has brought significant therapeutic effects for patients with cancers, including those with ESCC. Additionally, endoscopic screening plays a crucial role in the early diagnosis and treatment of esophageal cancer. Yet, due to the variability in the skill levels of endoscopists, many cases are still missed due to the inability to timely identify lesions (4, 5).

TME refers to the complex environment surrounding tumor cells, comprising intercellular interactions, extracellular matrix, vasculature, immune cells, and several other factors, all of which collectively influence tumor development, spread, and response to treatment (6–8). Within the TME, dendritic cells (DCs) serve as critical immune regulators, playing a vital role. Not only do they capture and present tumor antigens, but they also activate and modulate immune responses, impacting the mechanisms of tumor immune surveillance and escape (9). Previous studies have indicated that a combination therapy of pemetrexed and DCs as a third-line treatment for ESCC can significantly improve prognosis and is well-tolerated (10). Furthermore, in ESCC patients with regions rich in tertiary lymphoid structures, there is an increased infiltration of CD8+ T cells and DCs, which is associated with stronger anti-tumor immune activity (11).Specifically, conventional dendritic cells (cDCs) play a key role in cross-presenting tumor antigens and activating cytotoxic T cells; tolerogenic dendritic cells (tDCs) can modulate immune responses by inducing immune tolerance or generating regulatory T cells, thereby preventing overactive immune reactions (12); plasmacytoid dendritic cells (pDCs), primarily known for their role in antiviral responses, have an unclear role in the TME, possibly involving the modulation of the immune status of the TME (13). The functions and interactions of these three types of dendritic cells in tumor immunity provide a critical theoretical basis for the development of novel immunotherapeutic strategies.

Single-cell RNA sequencing(scRNA-seq) technology, in contrast to traditional Bulk-RNA sequencing, offers more precise and detailed analysis at the cellular level (14). scRNA-seq enables us to capture the heterogeneity within a cell population, revealing unique gene expression patterns of different cellular states and subgroups. This technology allows us to identify and differentiate rare cell types within a cell population, such as cancer or immune cells in tumors, thus offering new perspectives for understanding complex biological processes and disease mechanisms (15). Additionally, scRNA-seq can reveal interactions and communication pathways between cells, often unachievable in Bulk-RNA sequencing (16). Therefore, scRNA-seq not only enhances our understanding of biological systems at the microscopic level but also opens new doors for precision medicine and personalized treatment.

In our study, we utilized scRNA-seq to delve into signaling pathways associated with dendritic cells and conducted pseudotime analyses of three different types of dendritic cells: conventional cDC, pDC and tDC. Using the Copykat algorithm, we inferred the benign or malignant nature of these cells. We also carried out cell communication analyses between dendritic cells and other cell types within the single-cell sequencing data, revealing their interactions and functions in the tumor microenvironment. Additionally, in Bulk-RNA data, we analyzed the impact of these three types of dendritic cells on prognosis. Based on these findings, we constructed a model based on the marker genes of DC cells, which can accurately predict the prognosis and efficacy of immunotherapy in ESCC. To validate this model and the expression of its genes, we employed real-time quantitative PCR (qPCR) and immunohistochemistry techniques. The application of these methods not only further confirmed the accuracy of our model but also offered new strategies and targets for the personalized treatment of ESCC.

## 2 Methods

### 2.1 Data acquisition

This study obtained original scRNA-seq data of 9 cases of ESCC and 9 normal esophageal tissues from the National Center for Biotechnology Information Sequence Read Archive (SRA) (Project Number: PRJNA777911, URL: https://www.ncbi.nlm.nih.gov/sra/?term=PRJNA777911). Furthermore, we acquired dataset GSE53624 from the Gene Expression Omnibus (GEO), which includes sequencing data and clinical information of 119 ESCC samples and 119 normal samples, for the purpose of training our model. Subsequently, for model validation and further analysis, we obtained sequencing data (FPKM format), clinical information, and genetic mutation details of 198 ESCA patients from The Cancer Genome Atlas (TCGA) database (URL: https://portal.gdc.cancer.gov/repository). In order to better match the TCGA data with GEO data, we converted the gene expression information from FPKM format to TPM format. Moreover, aligning with previous studies, we retrieved information about 10 cancer-related biological pathways (17).

### 2.2 ScRNA-seq analysis

In the scRNA-seq analysis, we first utilized the hg38 reference genome and employed the default parameters of Cell Ranger v.7.1.0 software for gene alignment (18). Following this, we conducted standard single-cell RNA sequencing data analysis using the ‘Seurat’ package in R. After filtering out cells with mitochondrial gene percentage (pMT) over 20%, hemoglobin gene percentage (pHB) over 1%, and those expressing fewer than 500 genes, while retaining genes expressed in at least five cells, we successfully obtained 91,810 high-quality cell samples. Data normalization was achieved using the NormalizeData function, followed by the use of the FindVariableFeatures function to identify 2000 highly variable genes, and data scaling was accomplished through the ScaleData function. Next, Principal Component Analysis (PCA) was utilized for data dimensionality reduction. To further ensure comparability between different sequencing data, we employed the ‘Harmony’ R package to eliminate batch effects among samples (19). Utilizing t-distributed Stochastic Neighbor Embedding (t-SNE) technology, we visualized the above results, and ultimately identified 30 cell clusters. The annotation of each cell cluster was conducted by combining the ‘singleR’ package (20) and CellTypist (https://github.com/Teichlab/celltypist), based on the expression patterns of known marker genes. Moreover, we extracted DC cells using the subset function and subjected them to similar analytical processing as mentioned above. Subsequently, the analysis of intercellular communication was conducted using the ‘CellChat’ R package (21). Following this, we performed single-cell regulatory network inference and clustering (SCENIC) analysis on DC cells using the ‘Scenic’ R package (22) Additionally, the malignancy level of DC cells was inferred using the copykat R package (23), and pseudotime analysis was conducted with the ‘monocle2’ R package (24). Finally, we calculated marker genes between three types of dendritic cells using the FindallMarkers function, with selection parameters including logfc.threshold = 1, min.pct = 0.25, only.pos = T. Furthermore, the ‘SCP’ R package was used during the data visualization process.

### 2.3 Evaluation of dendritic cell-related features

In the GSE53624 cohort, enrichment scores for each type of DCs macrophage in every sample were calculated using the Single-sample Gene Set Enrichment Analysis (ssGSEA) algorithm (25), based on marker genes of dendritic cell. Initially, the differences in enrichment scores between normal and tumor samples were assessed, followed by dividing the ESCC tumor samples into two groups based on the median enrichment score, to conduct Kaplan-Meier survival analysis and assess survival differences between the groups.

### 2.4 Construction of the prognostic model

In this phase of our study, the ‘limma’ R package (26) was initially utilized to analyze the GSE53624 dataset, specifically aiming to identify differentially expressed genes (DEGs) between normal esophageal tissues and ESCC samples. The selection criteria were established as a false discovery rate (FDR) less than 0.05 and an absolute log2 fold change (|log2(FoldChange)|) greater than 1. After this evaluation, we analyzed the correlation between the DEGs and markers of three types of dendritic cells, selecting genes with a correlation coefficient greater than 0.4 and a p-value less than 0.05 for subsequent univariate Cox regression analysis to identify genes significantly impacting ESCC prognosis. Thereafter, a prognostic model was constructed using the identified genes in conjunction with least absolute shrinkage and selection operator (LASSO) Cox proportional hazards regression and multivariate regression. The model’s formula is: $\text{RiskScore}=\sum_{k=1}^{n}\;coef\left(k\right)\times Expr\left(K\right)$ where cof (k) is an abbreviation for the regression coefficient, and Expr (k) represents the expression level of the genes used in model construction. Based on the median risk score, all patients were categorized into high and low-risk groups. To enhance the confirmation of the model’s prognostic effectiveness, the ‘survivalROC’ R package was applied on two datasets: the training set GSE53624 and the validation set TCGA-ESCC. This was done to construct Receiver Operating Characteristic (ROC) curves and compute the Area Under the Curve (AUC). The aim of this procedure was to ascertain the risk model’s precision and evaluate its viability for clinical application.

### 2.5 Development of nomograms

Initially, both univariate and multivariate Cox regression analyses, integrating clinical features (such as age, alcohol consumption, lesion location, gender, and pathological staging) along with risk scores, were employed to identify factors significantly affecting ESCC prognosis. Utilizing these analysis outcomes, a nomogram was developed using the ‘rms’ package (27, 28) for predicting the survival probability of patients at 1, 3, and 5 years. To confirm the diagnostic and prognostic capabilities of the nomogram, decision curves and calibration curves were generated. These methods were utilized to assess the clinical benefits of the model at different risk thresholds and to evaluate the concordance between predicted survival probabilities and actual observed survival probabilities, thereby ensuring the accuracy and practicality of our model in clinical applications.

### 2.6 Analysis of mutations

Mutation data of ESCC patients were retrieved from the TCGA database using the ‘TCGAbiolinks’ R package and then uniformly decompressed. Using the read.maf function of the ‘maftools’ R package, mutation data and clinical information were read into MAF files. The oncoplot function was employed to create a heatmap that combines clinical and mutation information, showcasing the mutation profiles of high and low-risk groups, and the somaticInteractions function was used to analyze the co-mutation patterns of hub genes and the top 10 most frequently mutated genes in TCGA-ESCC.

### 2.7 Analysis of enrichment

Initially, the correlation between each Hub gene and common signaling pathways was assessed, followed by the application of the ‘GSEA’ algorithm (29) to analyze significantly enriched Gene Ontology (GO) pathways (30) and the Kyoto Encyclopedia of Genes and Genomes (KEGG) (31) between high and low-risk groups.

### 2.8 Forecasting the effectiveness of immunotherapy

Initially, we assessed the expression differences of immune checkpoint-related genes and major histocompatibility complex genes between high and low-risk groups. Subsequently, we validated the model’s prognostic and immunotherapy efficacy prediction capabilities in the IMvigor210 and GSE78220 cohorts, respectively.

### 2.9 RNA isolation and quantitative RT-PCR assay

Using TRIzol reagent from Thermo Fisher Scientific (Waltham, MA, USA), total RNA was extracted from ESCC cells and tissues. Following the protocol provided by the manufacturer, this RNA was then reverse-transcribed into complementary DNA (cDNA) using the RevertAid™ First Strand cDNA Synthesis Kit, also from Thermo Fisher Scientific. Quantitative real-time PCR (qRT-PCR) analyses were conducted using a Takara Bio’s SYBR Green PCR kit (Otsu, Japan) on Thermo Fisher Scientific’s StepOne Real-Time PCR system. For quantifying the levels of gene expression, the 2-△△CT method was employed.

### 2.10 Immunohistochemistry

Following approval by the Ethics Committee, 16 paraffin-embedded ESCC tissue sections, including tumors and corresponding peritumoral tissues, were obtained from the Pathology Department of the Second Hospital of Hebei Medical University. The immunohistochemical staining commenced with incubating the slides at 60°C for four hours to fix the tissue, followed by deparaffinization in xylene and rehydration through a graded series of alcohol. Subsequently, antigen retrieval was conducted in citrate buffer under high pressure for 20 minutes before the slides were cooled to room temperature and washed with PBS. Endogenous peroxidase activity was quenched with a 30-minute treatment of 3% hydrogen peroxide, followed by further PBS washes. The sections were then blocked with PBS containing 10% bovine serum. Primary antibody incubation proceeded overnight, after which the slides were washed with PBS, treated with secondary antibody for one hour, and washed again. Color development was achieved by applying DAB chromogen and timing the reaction carefully. Following this, the sections were rinsed with water, counterstained with hematoxylin for three minutes, washed again, and finally subjected to a dehydration, clearing, and mounting process. This sequence of steps completed the staining of the tissue sections for subsequent microscopic observation and analysis.

## 3 Results

### 3.1 Analysis of scRNA-seq

**Figure 1:**
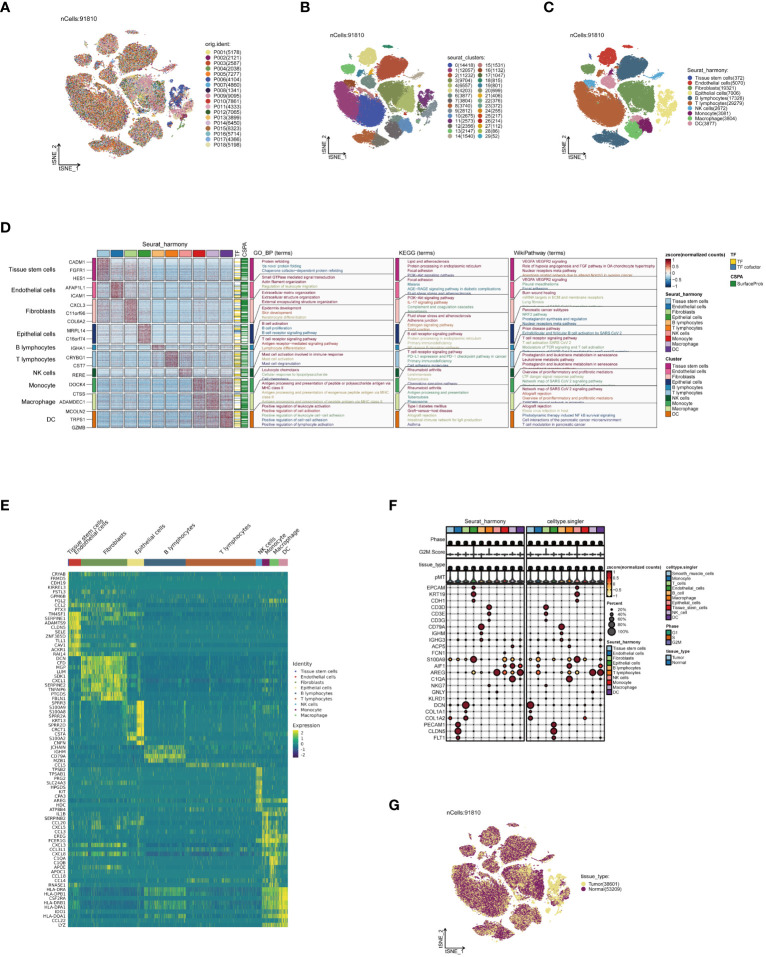
Annotation of single-cell data. **(A)** Distribution of single-cell data in a t-SNE plot, colored according to sample origin. **(B)** Thirty cell subpopulations obtained after dimensionality reduction clustering, each represented by a different color. **(C)** Following cell annotation, ten distinct cell subtypes were identified. **(D)** Enrichment analysis results of each cell subpopulation, with a heatmap showing unique gene expression patterns and activities in various biological pathways for different subpopulations. **(E)** Heatmap of the top 10 most highly expressed genes in all subpopulations, reflecting heterogeneity in expression among subpopulations. **(F)** Heatmap of marker gene expression among different cell subpopulations, revealing the accuracy of cell annotation. **(G)** t-SNE plot colored according to tissue origin.

Firstly, strict quality control measures were implemented on the data, resulting in the elimination of low-quality cells that did not meet the requirements (**Supplementary Figures 1A, B**). Subsequently, during the analysis phase, batch effects in the original dataset were identified, a critical factor when explaining differences between cells (**Supplementary Figure 1C**). Following this, the non-biological effects were effectively mitigated using the Harmony algorithm, thereby optimizing sample distribution and minimizing the impact of experimental condition variability on the analysis results (**Supplementary Figure 1D**). Thereafter, following data normalization and dimensionality reduction clustering, the single-cell data were visually depicted in a t-SNE plot. **Figure 1A**, depicting the cell distribution by sample origin, reveals that cells from different samples are evenly mixed in space, unaffected by significant batch effects. Continuing with the analysis, 30 cell subpopulations were identified from 91,810 high-quality cells, representing various cell states in the TME (**Figure 1B**). Through the integration of manual and algorithmic annotations, these subpopulations were further refined into 10 major cell clusters (**Figure 1C**). The functional annotation of these cell clusters highlighted that dendritic cells (DCs) are primarily associated with pathways such as positive regulation of leukocytes, allograft rejection, and T-cell regulation in pancreatic cancer (**Figure 1D**). **Figure 1E** presents the top 10 most highly expressed genes in each cell cluster, further elucidating the biological characteristics of each cluster. Furthermore, the expression patterns of marker genes in **Figure 1F**, **Supplementary Figure 1E** illustrate the precision of our clustering. Ultimately, **Figure 1G**, depicting the distribution of cell tissue origins, further confirms the uniform mixture of various cell types in ESCC, apart from epithelial cells, emphasizing the cellular heterogeneity in the TME.

### 3.2 Analysis of cell communication

**Figure 2:**
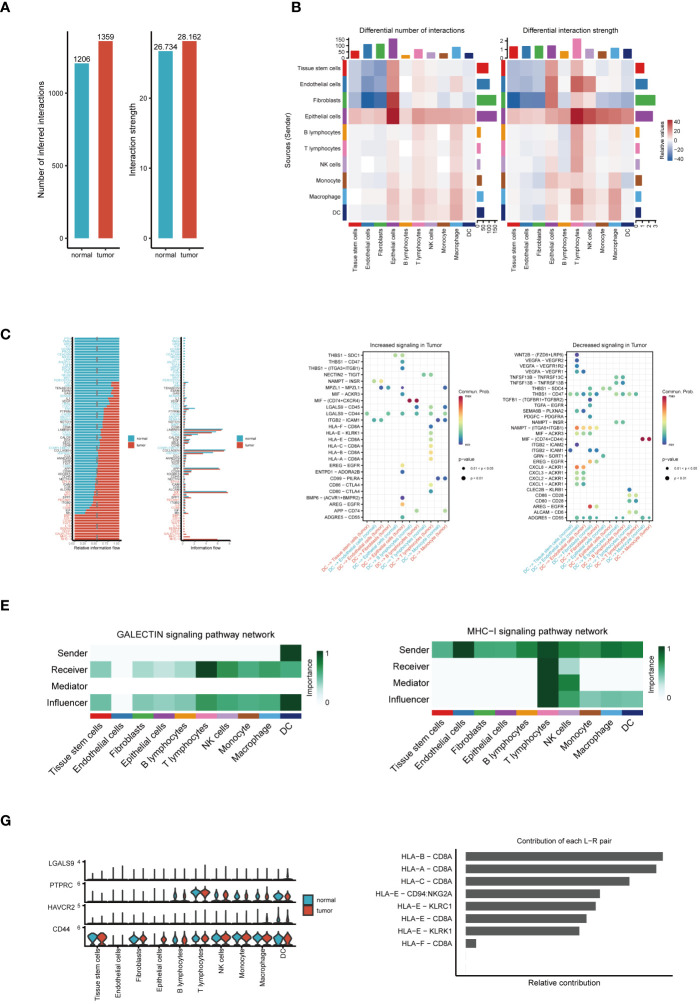
Analysis of cell communication. **(A)** Comparison of the number and intensity of cell communications between tumor and normal tissues. **(B)** Heatmap of communication probabilities between each cell subpopulation. **(C)** Differences in the number and intensity of signaling pathway-related cell communications between tumor and normal tissues. **(D)** Key ligand-receptor pairs with significant changes in tumor tissues. **(E)** The Function of the GALECTIN Signaling Pathway in Cellular Interaction. **(F)** The Importance of the MHC-I Signaling Pathway in Intercellular Communication. **(G)** Expression differences of molecules in the GALECTIN pathway between tumor and normal samples. **(H)** Relative contribution of receptor-ligand pairs in the MHC-I pathway.

Our cellular communication analysis revealed a marked escalation in both the number and intensity of intercellular communications within tumor tissues, as opposed to normal tissues, with **Figure 2A** visually supporting this finding. This trend likely indicates a heightened level of interaction between tumor cells and adaptive responses within the TME. Elaborating on these insights, the heatmap in **Figure 2B** uncovers noticeable disparities in communication probabilities among different tumor cell subpopulations, particularly highlighting the intensified interactions between dendritic and epithelial cells in tumor contexts. Delving into signal pathway analysis, illustrated in **Figure 2C**, we discerned notable variances in the frequency and intensity of cellular communications between tumor and normal tissues. This analysis brought to light the prominent activation of pathways such as GALECTIN, MHC−I, and MHC−II within tumors, hinting at their potential pivotal roles in tumor evolution. Following this trajectory, our subsequent examination, as demonstrated in **Figure 2D**, brought to the fore significant alterations in ligand-receptor pairs within tumor tissues. These specific molecular interactions suggest critical regulatory roles in intercellular communication, thereby unveiling potential new molecular targets for therapeutic intervention. A deeper dive into the GALECTIN and MHC-I pathways’ roles in cellular communication revealed distinct dynamics. The GALECTIN pathway, predominantly initiated by dendritic cells within the TME and targeting T lymphocytes, is illustrated in **Figure 2E**. Conversely, the MHC−I pathway, prominently active in T lymphocytes and functioning as a signal emitter in dendritic cells, is depicted in **Figure 2F**. Intriguingly, the comparative analysis of key molecules in the GALECTIN pathway between tumor and normal samples, as seen in **Figure 2G**, did not indicate significant expression differences. This suggests that their role in the TME might be mediated through mechanisms beyond mere expression level alterations. Concluding our analysis, **Figure 2H** elucidates the pivotal receptor-ligand pairs in the MHC-I pathway, particularly highlighting the dominance of HLA-B - CD8A, HLA-A - CD8A, and HLA-C - CD8A in tumor communication. This finding accentuates their significance in the tumor immune landscape, potentially positioning them as central targets for future therapeutic exploration. These comprehensive analytical results not only demonstrate the intricacies and dynamic nature of cellular communication within the TME but also pinpoint potential key regulatory nodes in tumor progression. This provides novel insights for developing ESCC treatment strategies and lays a solid foundation for future advancements in precision medicine research.

### 3.3 Further clustering of dendritic cells

**Figure 3:**
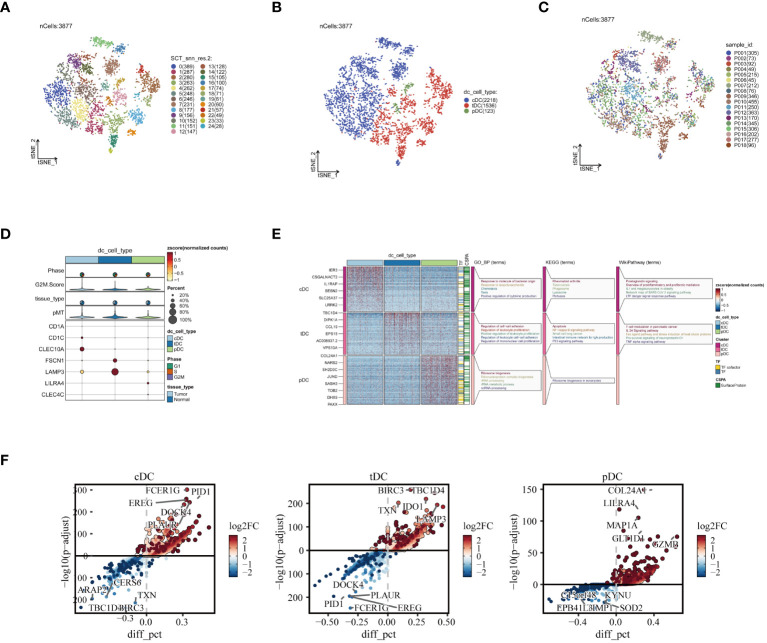
Reclustering of DC cells. **(A)** DCs divided into 25 cell subpopulations after dimensionality reduction clustering, each represented by a different color. **(B)** Dendritic cells annotated as three different cell types based on phenotype. **(C)** t-SNE plot showing the origins of each DCs sample, each identified by a unique color. **(D)** Illustration of dendritic cell marker gene expression. **(E)** Enrichment analysis results, with a heatmap revealing the activity of different dendritic cell subpopulations in various biological pathways and functions. **(F)** Presentation of differential genes in three types of dendritic cells, with scatter plots revealing changes in their expression levels and potential significance in tumor biology.

In order to gain a deeper understanding of DC subgroups, we initially utilized the subset function within the Seurat package, specifically focusing on isolating DC cells for enhanced scrutiny. This approach led to the identification of 3,877 high-quality DC cells following the crucial steps of data normalization and dimensionality reduction clustering. These cells were distinctly visualized in a t-SNE plot, where they were further subdivided into 25 unique subgroups, as illustrated in **Figure 3A**. Subsequently, by employing specific marker genes for cell annotation (32), we meticulously differentiated these cells into three DC subtypes: cDCs, tDCs, and pDCs. Each subtype, with its unique features, is concisely represented in **Figure 3B**. Furthermore, the analysis of the sample origin distribution of these DC cells, showcased in **Figure 3C**, revealed a uniform distribution across various samples. This uniformity is indicative of minimal batch effect influence, thereby reinforcing the reliability of our clustering approach. Advancing further into our investigation, the expression analysis of marker genes was conducted, as depicted in **Figure 3D**. This analysis not only validated the precision of our cell clustering but also enriched our understanding of the cellular characteristics. Moreover, the functional enrichment analysis of the three DC subgroups, detailed in **Figure 3E**, unveiled distinct functional pathways associated with each subgroup. In **Figure 3F**, we demonstrate the significantly overexpressed and underexpressed differential genes across three dendritic cell types. We discovered that cDC cells are predominantly involved in pathways like ‘response to molecule of bacterial origin’ and ‘positive regulation of cytokine production’, while tDC cells are linked to the ‘regulation of leukocyte proliferation’. In contrast, pDC cells are primarily associated with ‘ribonucleoprotein complex biogenesis’. These insights elucidate the diverse and significant roles that different DC subgroups play within the immune system, further contributing to our comprehensive understanding of their functionalities in various physiological contexts.

### 3.4 Pseudotime analysis and assessment of heterogeneity among samples

**Figure 4:**
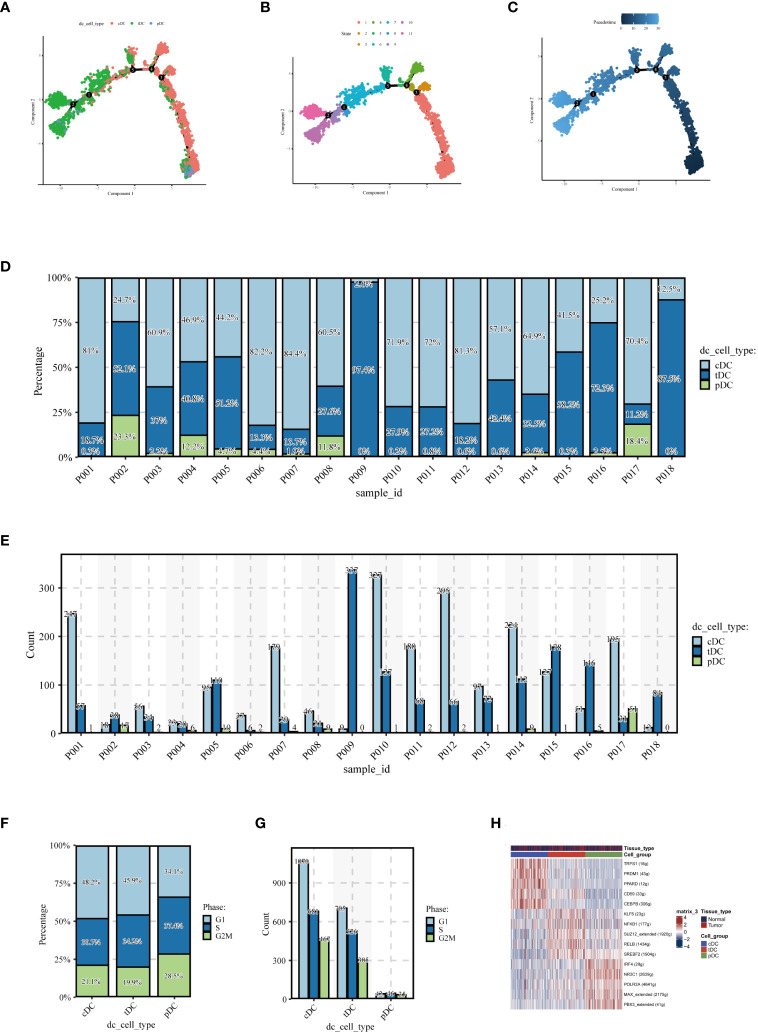
Pseudotime analysis and cell proportion analysis. Pseudotime analysis: **(A)** colored according to cell type, **(B)** colored according to cell state, **(C)** colored according to developmental time. **(D)** Stacked bar graph showing the relative proportions of cDC, tDC, and pDC cell types in different samples, reflecting the heterogeneity of dendritic cell composition among samples. **(E)** Each bar represents the number of different dendritic cell types in a sample, providing a visual comparison of the numbers of cDC, tDC, and pDC cells in each sample. **(F)** Proportion of the cell cycle in each type of dendritic cell. **(G)** Number of cells in different cell cycle phases for each type of dendritic cell. **(H)** Heatmap displaying transcription factors that may regulate the three types of dendritic cells.

To elucidate the evolutionary relationships among the three types of DCs, we embarked on a pseudotime analysis using the Monocle2 package. This analysis revealed that DC cells progress through nine distinct differentiation states. Intriguingly, cDCs were predominantly found in the early stages of differentiation, while tDCs appeared to advance towards later stages. pDCs, however, were present in both early and late stages, as illustrated in **Figures 4A-C**. This finding points towards the dynamic and complex nature of DC cell differentiation. Further examining the number and percentage of DC cells among different patients, as shown in **Figures 4D, E**, we uncovered significant heterogeneity. This variability suggests that DC cells may assume diverse roles in different individuals, highlighting the complexity of their functions in the immune response. Subsequently, we compared the cell cycle distribution of the dendritic cell subpopulations, presented in **Figures 4F, G**. This comparison revealed that pDCs had a higher proportion of cells in the G2/M and S phases, indicating a more active cell cycle status, whereas cDCs and tDCs predominantly occupied the G1 phase. Delving deeper, our analysis, as demonstrated in **Figure 4H**, focused on the expression patterns of various transcription factors across dendritic cell types. A detailed examination unveiled distinct expression trends for transcription factors such as IRF1, NFKB1, and RELB within the DC subgroups. Notably, IRF1 expression was markedly higher in cDC cells compared to pDC cells. NFKB1 exhibited relatively high expression in both cell types, albeit more pronounced in cDCs. Conversely, an increased expression of RELB was observed predominantly in pDC cells. Additionally, POLR2A was found to be highly expressed across all dendritic cell types, particularly in cDC cells, which might indicate its broad and pivotal role in dendritic cell functions. These results shed light on potential transcriptional regulatory differences between dendritic cell subgroups, which are integral to comprehending their distinct functions in immune responses. The unique expression patterns of these transcription factors likely mirror the specialized roles of dendritic cells in immune surveillance, antigen presentation, and inflammatory responses, thereby contributing to our understanding of the intricate dynamics within the immune system.

### 3.5 Impact of dendritic cell subgroups on ESCC prognosis

**Figure 5:**
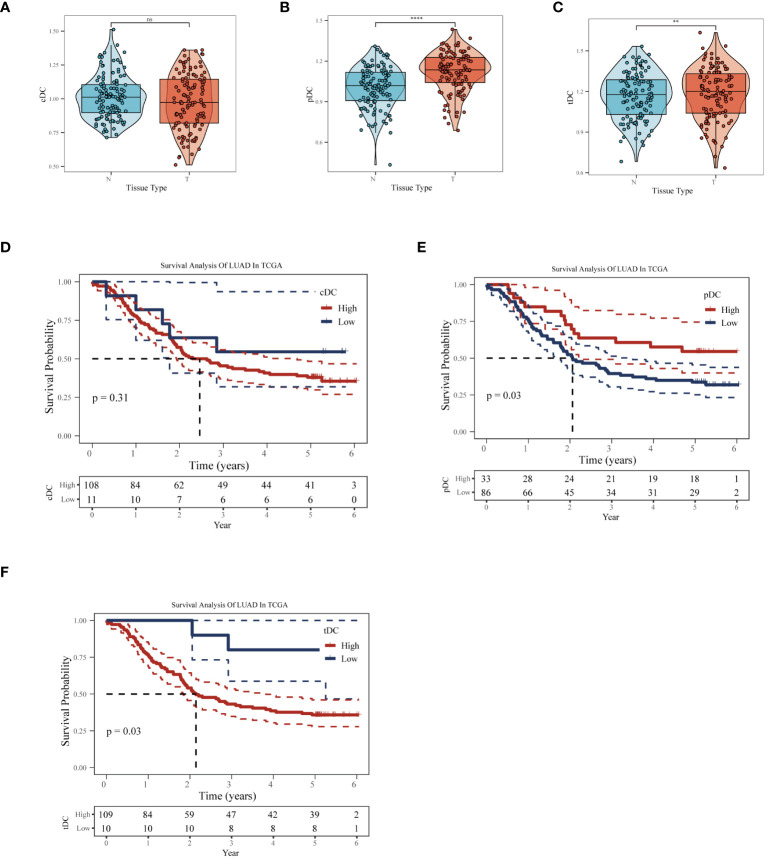
Impact of dendritic cell subgroups on ESCC prognosis. In the GSE53624 dataset, **(A)** cDC, **(B)** tDC, and **(C)** pDC GSVA enrichment scores comparison between normal esophagus and ESCC tissues. **(D)** Kaplan-Meier survival curves for cDC, **(E)** tDC, and **(F)** pDC, used to assess the correlation between these dendritic cell subgroup enrichment scores and the prognosis of esophageal squamous cell carcinoma patients. **P < 0.01, ****P < 0.0001.

In the GSE53624 dataset, we conducted a thorough analysis of the differences in the behavior of cDC, tDC, and pDC dendritic cells between normal esophageal tissue and ESCC tissue. **Figures 5A-C** revealed significant differences in the enrichment scores of the three types of DCs between normal and cancerous tissues. In particular, tDC and pDC showed significantly higher enrichment scores in ESCC tissues compared to normal tissues, reflecting their possible activated state in the TME. Further survival analysis using the Kaplan-Meier curve method explored the correlation between these enrichment scores and patient prognosis. **Figures 5D, E** showed that high levels of cDC and tDC enrichment fractions were positively correlated with worse prognosis in ESCC patients. While the correlation for cDC did not reach statistical significance (p=0.31), tDC exhibited a statistically significant correlation (p=0.03).In stark contrast, high levels of pDC enrichment scores were significantly correlated with better prognosis in patients (**Figure 5F**). These results revealed the unique roles of different dendritic cell subgroups in the pathogenesis of esophageal cancer and their potential mechanisms affecting patient prognosis. The significant association of tDC and pDC enrichment scores with patient survival probabilities highlights their importance as potential biomarkers in the TME and in future clinical decision-making. These findings provide valuable molecular targets for future therapeutic strategies targeting dendritic cells and offer new perspectives for clinical prognosis assessment.

### 3.6 Dendritic cell malignancy inference

**Figure 6:**
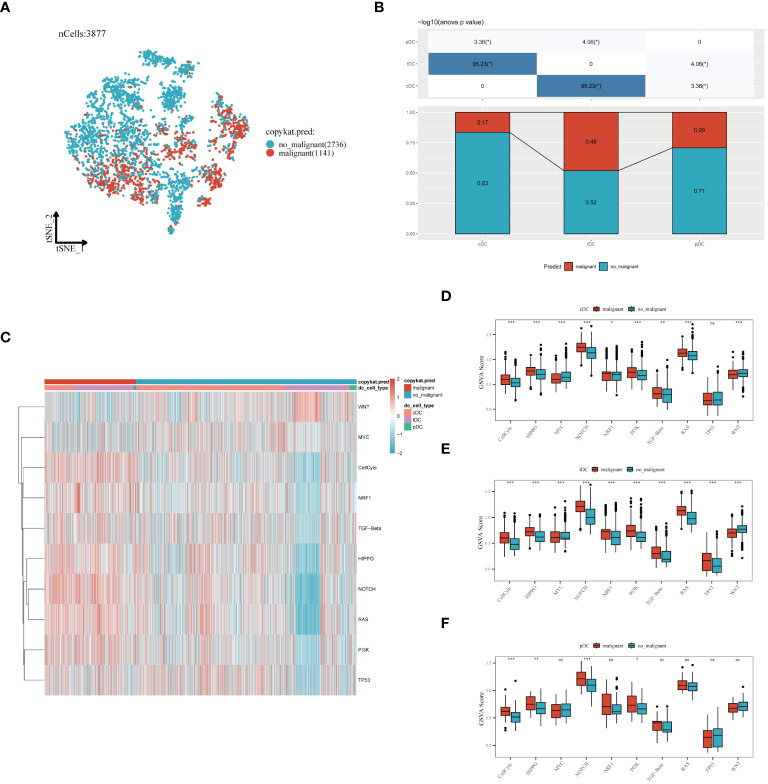
Evaluation of dendritic cell subgroups in benign and malignant classification, signal pathway correlation, and functional status. **(A)** t-SNE plot generated using the Copykat algorithm to infer benign and malignant states in dendritic cells. B) Comparison of the proportion of malignant cells in three types of dendritic cells (cDC, tDC, pDC). **(C)** Heatmap showing the correlation of three dendritic cell subpopulations with ten tumor-related signaling pathways. **(D)** cDC, **(E)** tDC, **(F)** pDC: Differences between benign and malignant cells in tumor-related signaling pathway scores (GSVA enrichment scores). *P < 0.05, **P < 0.01, ***P < 0.001.

In our in-depth molecular characterization of DC subgroups, we employed the Copykat algorithm to estimate the benign or malignant state of each cell. t-SNE visualization results indicated a clear distinction between malignant and benign cells among the 3,877 cells analyzed, with 1,141 malignant and 2,536 benign cells identified (**Figure 6A**). Comparing the proportion of malignant cells across three different types of dendritic cells revealed variability in malignancy rates within each cell type, potentially reflecting their distinct functions or pathological states in the TME (**Figure 6B**). Specifically, cDCs had the lowest proportion of malignancy (17%), followed by pDCs (29%), and tDCs had the highest (48%). The activity of ten tumor-related signaling pathways was assessed using GSVA, with the results presented in a heatmap format (**Figure 6C**). This analysis demonstrated a more significant association of malignant cells with these pathways, revealing specific correlations between each dendritic cell type and certain signaling pathways, providing clues to their potential roles in tumor development. Further analysis of functional state differences between benign and malignant cells within each subgroup showed significant scoring differences in multiple tumor-related signaling pathways for cDCs (**Figure 6D**), tDCs (**Figure 6E**), and pDCs (**Figure 6F**). This disparity may reveal different mechanisms by which malignant dendritic cells promote tumor growth and modulate tumor immune responses. Integrating these findings, we conclude that different dendritic cell subgroups exhibit distinct signaling pathway activation patterns and functional states in malignant tumors, which is crucial for understanding their roles in the tumor immune microenvironment.

### 3.7 Development and assessment of the predictive mode

**Figure 7:**
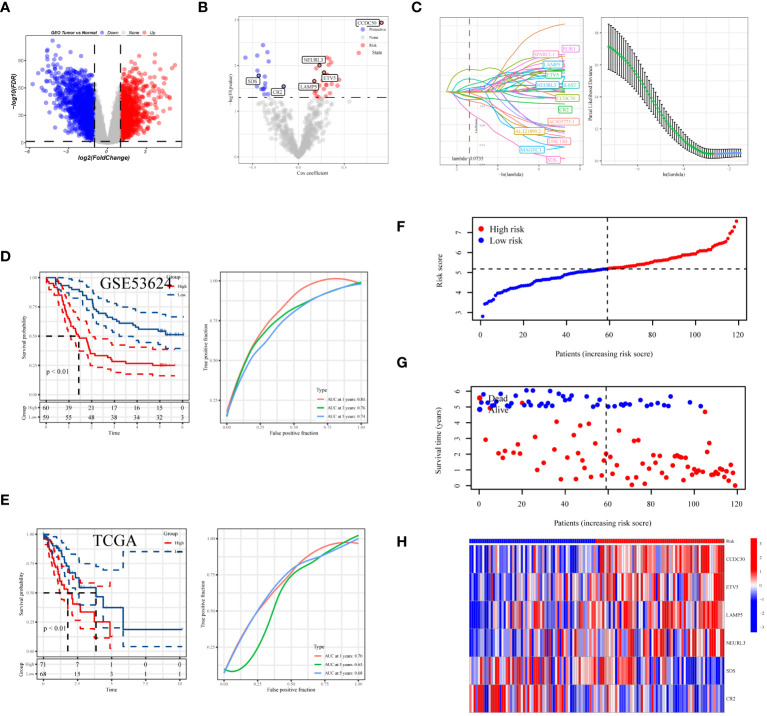
Development and assessment of the predictive model. **(A)** Volcano plot showing the distribution of differentially expressed genes in the GSE53624 dataset, with red indicating upregulated genes, blue indicating downregulated genes, and size representing the significance of gene expression changes. **(B)** Results of univariate regression analysis of dendritic cell-related genes. **(C)** Optimal prognostic markers identified via Lasso regression analysis and their respective coefficient shrinkage trajectories. **(D)** Kaplan-Meier survival curves for ESCC patients in the GSE53624 and **(E)** TCGA datasets (left), and receiver operating characteristic (ROC) curve analysis of the prognostic model at 1 year, 3 years, and 5 years (right). **(F)** Risk score distribution plot differentiating between high and low-risk statuses of patients. **(G)** Distribution plot of survival statuses illustrating survival scenarios of patients in high and low-risk scoring categories. **(H)** Heatmap providing a detailed display of HUB gene expression in different samples.

In the process of conducting an in-depth analysis of the GSE53624 dataset, we initially calculated the differentially expressed genes (DEGs), selecting genes with an absolute log fold change (|lgFC|) greater than 1 and a p-value less than 0.05 (**Figure 7A**). Next, we analyzed the correlation between tDC and pDC marker genes and these DEGs, selecting genes with a correlation coefficient greater than 0.4 and a p-value less than 0.05. Based on these genes, univariate Cox regression analysis identified 46 genes with significant prognostic value for esophageal squamous cell carcinoma (ESCC), of which 28 were risk factors and 18 were protective factors (**Figure 7B**). To further narrow down the gene pool, we employed Lasso regression analysis and selected 13 genes at the optimal cutoff value (lambda=0.0735) (**Figure 7C**). Ultimately, we constructed a prognostic model comprising six genes using stepwise multivariate Cox regression, including Coiled-Coil Domain Containing 50 (*CCDC50*), ETS Variant 5 (*ETV5*), Neuralized E3 Ubiquitin Protein Ligase 3 (*NEURL3*), Lysosomal-Associated Membrane Protein Family Member 5 (*LAMP5*), Complement Receptor 2 (*CR2*), and Serine Dehydratase (*SDS*). The model formula is: Risk = 0.684 * *CCDC50 +* 0.221 * *ETV5 +* 0.158 * *LAMP5 +* 0.203 * *NEURL3* - 0.62 * *SDS* - 0.15 * *CR2*. Kaplan-Meier survival analysis was used to evaluate the prognosis of patients in high-risk and low-risk groups, with results indicating a significantly worse prognosis for the high-risk group. In the training set, the prognostic model demonstrated good predictive performance with area under the curve (AUC) values of 0.81, 0.76, and 0.74 for 1-year, 3-year, and 5-year survival predictions, respectively (**Figure 6D**). Additionally, we validated the prognostic assessment and diagnostic capabilities of the model in the TCGA-ESCA dataset, obtaining similar positive results (**Figure 7E**). The risk score distribution chart (**Figure 7F**) clearly differentiated between high-risk and low-risk group patients, while the survival status distribution chart (**Figure 7G**) showed a noticeably higher number of deaths in the high-risk group compared to the low-risk group. Lastly, a heatmap (**Figure 7H**) detailed the expression patterns of the six core genes in the training set, further supporting their critical roles in esophageal cancer. In summary, the gene signature model we constructed is not only statistically significant but also holds potential clinical application value. It provides important molecular markers for the personalized treatment of esophageal cancer patients.

### 3.8 Building and testing of the prognostic nomogram

**Figure 8:**
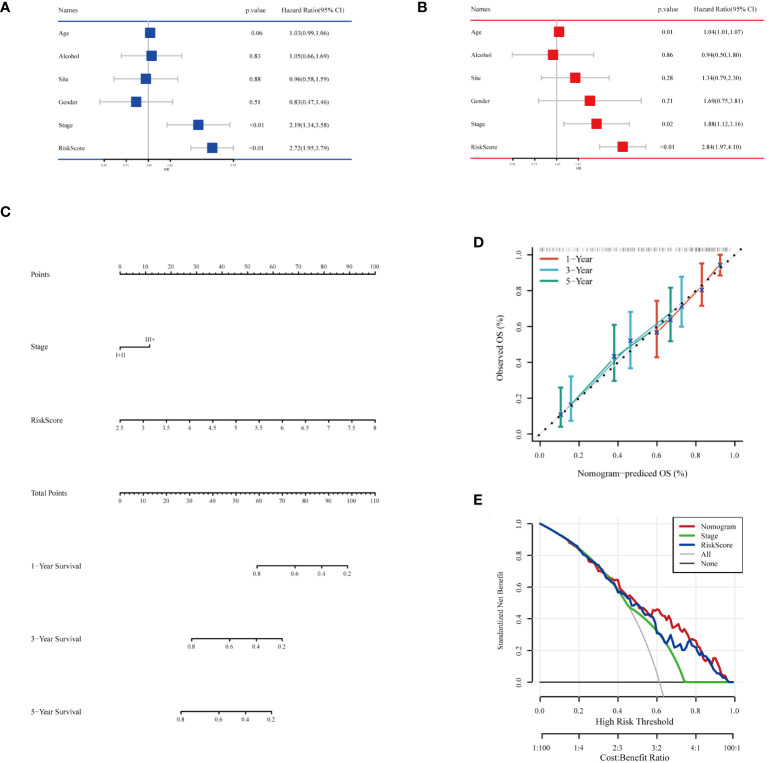
Building and testing of the prognostic nomogram. **(A)** Results of univariate Cox regression analysis combining clinical features and risk scores. **(B)** Results of multivariate Cox regression analysis combining clinical features and risk scores. **(C)** Nomogram constructed based on multivariate Cox regression analysis results, used to calculate the total score based on patients’ clinical features and risk scores, subsequently predicting 1-year, 3-year, and 5-year survival probabilities. **(D)** Calibration curves showing the concordance between the model’s predicted survival probabilities and the actual observed survival rates, providing validation for the 1-year, 3-year, and 5-year survival rate predictions. **(E)** Decision Curve Analysis (DCA) indicating the clinical value of the model at different risk thresholds by comparing net benefits when including different variable combinations, assessing the practical application benefits of the model.

To comprehensively assess the prognosis of ESCC patients and its applicability in clinical decision-making, we conducted a series of statistical analyses and model validations. Initially, we identified factors significantly affecting the prognosis of ESCC patients through univariate and multivariate Cox regression analyses, combined with clinical features and risk scoring (**Figures 8A, B**). The analysis indicated that pathological staging (Stage) and risk scoring are important factors affecting prognosis. Based on these findings, we constructed a nomogram (**Figure 8C**) combining risk scores and pathological staging to predict 1-year, 3-year, and 5-year survival probabilities. This tool aims to provide a quantitative method to assist physicians in treatment decision-making, estimating patients’ survival probabilities by calculating a total score for each patient. To validate the predictive accuracy of this nomogram, we plotted calibration curves (**Figure 8D**) to assess the concordance between the predicted and actual survival probabilities. The calibration curves demonstrated the model’s accuracy in predicting 1-year, 3-year, and 5-year survival, providing an intuitive validation of the model’s predictive capability. From the calibration curves in **Figure 8D**, our nomogram prediction model demonstrated good accuracy in forecasting 1-year, 3-year, and 5-year survival probabilities of ESCC patients in the training set. The calibration curves closely followed the ideal line, indicating a match between predicted survival probabilities and actual observed survival rates, confirming good calibration performance of the model at different time points. In the decision curve analysis, when applying our nomogram prediction model, particularly at moderate threshold ranges, the model showed higher net benefits, indicating strong clinical applicability in differentiating medium to high-risk groups of ESCC patients (**Figure 8E**). This result emphasizes the important value of the model in accurately stratifying patient risk and assisting in the formulation of corresponding treatment strategies. Overall, our analysis revealed a powerful prognostic assessment tool that combines clinical features and biomarker scoring of patients, effectively predicting the survival probabilities of ESCC patients. The model’s accuracy and clinical utility in predicting the prognosis of esophageal cancer patients were confirmed through the validation by calibration curves and decision curve analysis.

### 3.9 Enrichment analysis

**Figure 9:**
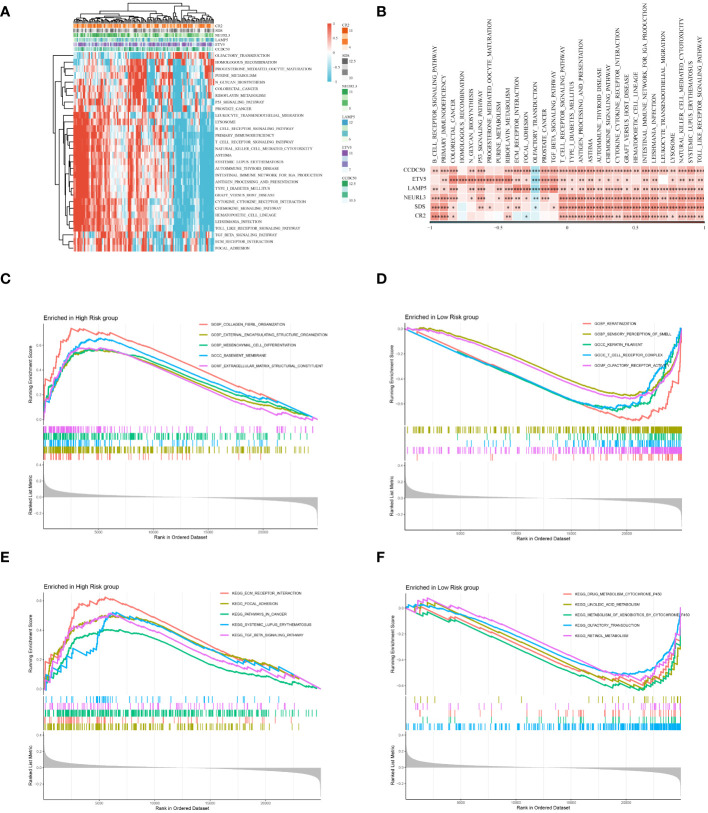
Enrichment analysis. **(A)** Displays a heatmap of enrichment scores for key pathways, visualizing the extent of enrichment in various samples. **(B)** Reveals the correlation between Hub genes and key pathways. **(C)** Results of the GO enrichment analysis for the high-risk group. **(D)** Results of the GO enrichment analysis for the low-risk group. **(E)** Results of the KEGG enrichment analysis for the high-risk group. **(F)** Results of the KEGG enrichment analysis for the low-risk group. *P < 0.05, **P < 0.01, ***P < 0.001.

An in-depth bioinformatics analysis of gene expression data from ESCC patients revealed significant differences in biological processes and metabolic pathways between different risk groups, offering new perspectives on the molecular characteristics and pathological mechanisms of these patient groups. As shown in **Figures 9A, B**, we first analyzed potential signaling pathways related to the genes used in model construction. A total of 30 pathways were significantly related to these genes, including the B cell receptor signaling pathway, primary immunodeficiency, colorectal cancer, etc., which play key roles in the biology and clinical characteristics of tumors. Subsequent GO and KEGG enrichment analyses further emphasized the molecular-level differences between different risk groups. As illustrated in **Figure 9C**, GO enrichment analysis showed that gene expression features of patients in the high-risk group were primarily focused on processes related to tumor invasiveness and metastasis, such as external encapsulating structure organization and collagen fibril organization. This finding suggests that tumors in these patients may have a stronger tendency for invasiveness and deterioration. In contrast, the low-risk group showed enrichment in processes related to immune response, such as keratinization and T cell receptor complex (**Figure 9D**), possibly reflecting a stronger immune response and lower tumor invasiveness in these patients. KEGG enrichment analysis results (**Figures 9E**, **F**) further highlighted these differences. The high-risk group was significantly enriched in ecm receptor interaction and pathways in cancer, suggesting that the tumor microenvironment might be more conducive to tumor growth and spread. Conversely, the low-risk group was mainly enriched in drug metabolism-cytochrome P450 and linoleic acid metabolism pathways, revealing unique characteristics of these patients in drug and lipid metabolism. In summary, our analysis disclosed marked differences in molecular traits and biological processes between high-risk and low-risk groups among ESCC patients.

### 3.10 Forecasting the effectiveness of immunotherapy

Initially, we compared the expression differences between immune checkpoint-related genes and major histocompatibility complex genes in high and low-risk groups (**Supplementary Figures 2A, B**). The results indicated that these genes, including classic molecules like PDCD1 and HAVCR2, were predominantly expressed at higher levels in the low-risk group. Next, we compared the survival differences between high and low-risk groups in the IMvigor210 cohort. Consistent with previous analyses, there were significant prognostic differences between the different risk groups (**Supplementary Figure 2C**). Additionally, in the group with better immunotherapy responses (CR+PR group), there were lower risk scores (**Supplementary Figure 2D**). Concurrently, the proportion of patients with poorer immunotherapy responses (PD+SD group) was significantly lower in the low-risk group (**Supplementary Figure 2E**). Lastly, we validated the prognostic ability of the model in another immunotherapy cohort, obtaining similar results (**Supplementary Figures 2F-H**).

### 3.11 Validation of model gene expression

**Figure 10:**
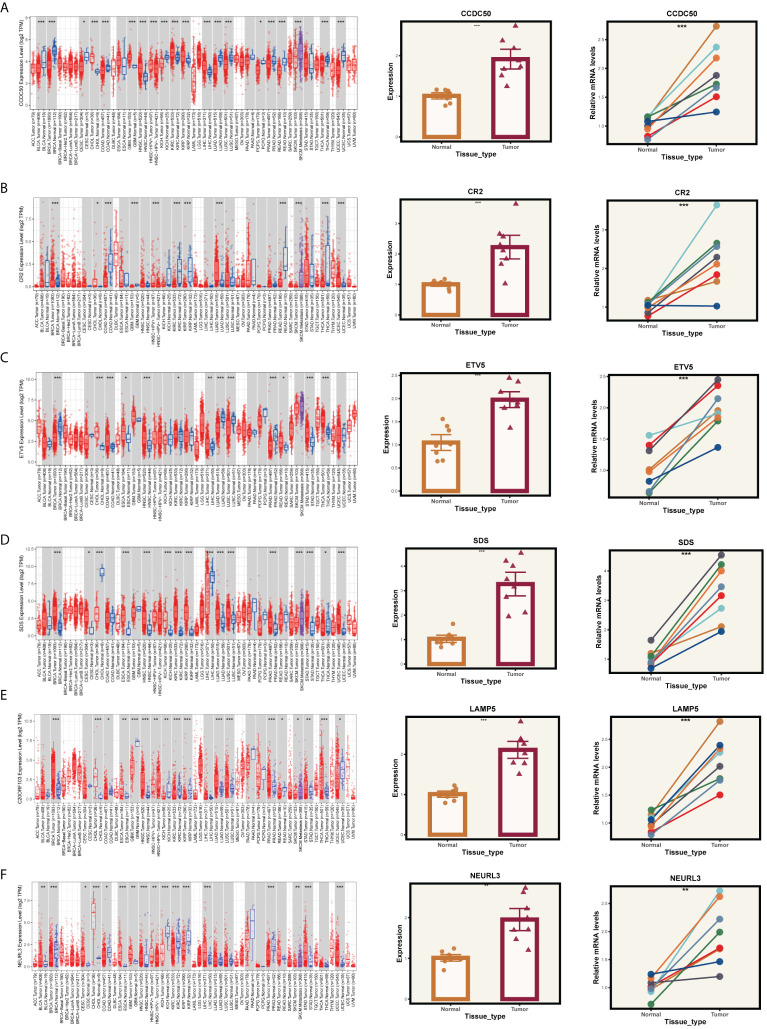
PCR validation of gene expression. Expression of genes **(A)** CCDC50, **(B)** CR2, **(C)** ETV5, **(D)** SDS, **(E)** LAMP5, and **(F)** NEURL3 in tumor and normal tissue samples. The left panels show the gene expression profiles across pan-cancer. The middle panels depict the comparative expression of these genes in tumor versus normal tissues. The right panels present paired comparisons between individual tumor tissues and their adjacent normal tissues. *P < 0.05, **P < 0.01, ***P < 0.001.

**Figure 11:**
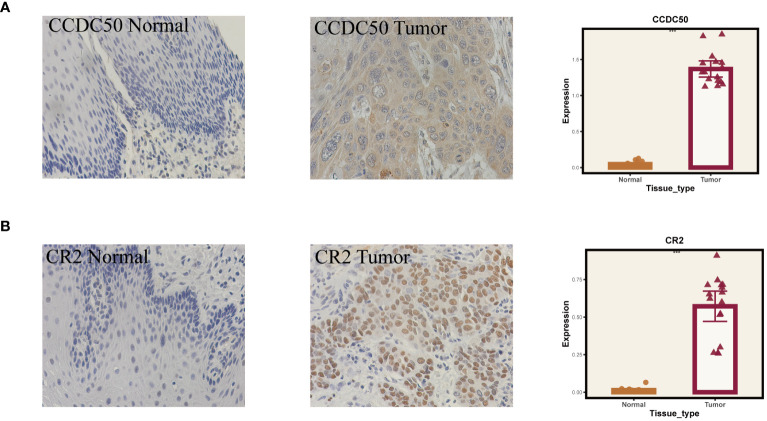
Immunohistochemical validation of gene expression. **(A)** Results for CCDC50. **(B)** Results for CR2. On the left are normal tissues, in the middle are tumor tissues, and on the right are the statistical results. ***P < 0.001.

Given that our analysis was primarily based on bioinformatics, it is possible that certain biases existed. To confirm the accuracy of our analysis, we initially downloaded the expression data of six model genes across various cancers from the Timer2.0 database. We then conducted qPCR and IHC validations of these model genes’ expression levels. As shown in **Figure 10**, our qPCR analysis (involving 8 pairs of ESCC patients and their corresponding peritumoral tissues) revealed significant overexpression of the six model genes in tumor tissues. While the pan-cancer analysis did not demonstrate statistical significance for CCDC50 and CR2, the trend of gene overexpression was consistent. This discrepancy might be attributed to the genetic differences between Eastern and Western populations. We further validated the expression of CCDC50 and CR2 at the protein level using IHC, which confirmed their significant overexpression in tumor tissues (**Figure 11**). These analyses substantiate the accuracy and credibility of our bioinformatics finding.

## 4 Discussion

ESCC is a globally prevalent disease, occupying a leading position in both incidence and mortality among malignancies (1). Traditional treatments such as surgery, chemotherapy, and radiotherapy, augmented by a limited range of targeted therapies, have been the mainstay. However, the advent of emerging strategies like immunotherapy has injected a ray of hope into the realm of ESCC management (3). Despite these advancements, the battle against ESCC is fraught with challenges, primarily due to the inherent difficulties in early diagnosis and the multitude of complex postoperative complications. Accurate molecular characterization is imperative for effectively targeting ESCC.

In solid tumors, especially ESCC, the significance of TME in influencing cancer therapy has garnered escalating attention. The microenvironment of ESCC, a complex milieu comprising diverse cellular groups, signaling molecules, and structural components, facilitates an intricate interplay with cancer cells, thereby supporting every phase of tumor development (33). Environmental factors, for instance, can instigate chronic inflammation, perpetuating pro-inflammatory signaling pathways that bolster tumor cell survival and proliferation (34). The anti-tumor immune response is often undermined by elements such as myeloid-derived suppressor cells, regulatory T cells, and immune checkpoints like programmed death-1 (35). Moreover, tumor-associated macrophages and other immune cells can assume additional tumor-promoting roles, including the induction of angiogenesis and facilitation of tumor cell invasion (36). Furthermore, cancer-associated fibroblasts secrete growth factors and modify the extracellular matrix, crafting a conducive tumor niche that accentuates tumor cell migration and metastasis (37).

Notably, dendritic cells (DCs), as professional antigen-presenting cells, play a pivotal role in the tumor microenvironment. These cells adeptly capture exogenous antigens and present them to lymphocytes, such as T and B cells, epitomizing one of the most potent cells in triggering the adaptive immune response (38). Consequently, the presence of DCs at immune challenge sites is essential for mounting an effective immune response. A significant area of current research is how different tumor microenvironments influence DCs in aspects like their development, functionality, and migration, thereby modulating the robustness of the adaptive immune response. Given their heterogeneity, DCs are classified into various subtypes, each with distinct functionalities. In our study, we categorize DCs into three subtypes - cDC, tDC, and pDC - in alignment with established literature. Our ssGSEA analysis reveals that pDCs positively influence ESCC prognosis, while a high enrichment of tDC-related genes correlates with poorer outcomes in ESCC patients. Through intercellular communication analysis, we have pinpointed GALECTIN and MHC−I as potential carcinogenic pathways in these DC subsets. GALECTINs, crucial in cancer progression, play unique roles in the tumor microenvironment by modulating tumor cell adhesion, migration, invasion, and impacting immune cell functions (39). Consequently, GALECTIN inhibitors or modulators could emerge as innovative therapeutic approaches in cancer treatment, potentially improving patient outcomes. The aberrant regulation of MHC-I molecules in cancer, possibly exploited by tumor cells to evade immune detection and promote tumor progression, diminishes the efficacy of cancer immunotherapies. The interactions of MHC-I molecules within the tumor microenvironment and their multifaceted roles in cancer progression are currently at the forefront of oncological research (40). Targeted therapies against MHC-I hold substantial promise in enhancing the effectiveness of cancer immunotherapies and in deepening our understanding of the dynamic roles and mechanisms of MHC-I in cancer.

During our examination of the cell cycle distribution across different dendritic cell (DC) subgroups, notable variances were found in the proportions of cell cycle stages among diverse types of DCs. Specifically, pDCs exhibited a higher proportion of cells in the G2/M and S phases, suggesting a more active state of cell division or preparation for cell division. In contrast, cDCs and tDCs predominantly resided in the G1 phase, indicating a relatively quiescent state in the cell cycle. This distribution pattern of cell cycle states may reflect the distinct biological functions and activities of each cell type within the tumor microenvironment. Notably, the increased proportion of pDCs in the G2/M phase could be associated with their role in viral defense and tumor immune surveillance, whereas the dominance of cDCs and tDCs in the G1 phase might align with their functions in antigen presentation and maintaining immune tolerance. These findings provide cell cycle-related insights for further exploration of the roles of DCs in tumor development and immune responses.

Based on six DC marker genes (CCDC50, ETV5, LAMP5, NEURL3, SDS, CR2), we constructed a robust prognostic feature set that can reliably predict the prognosis and efficacy of immunotherapy in ESCC. CCDC50, a gene encoding a human protein, has been explored in multiple studies for its primary functions and mechanisms. Its increased expression in diffuse large B-cell lymphoma (DLBCL) has been linked to tumor development stages and extranodal site numbers. Additionally, CCDC50 promotes tumor cell proliferation by inhibiting c-Myc ubiquitin-mediated degradation (41) and also contributes to the development of hepatocellular carcinoma through the Ras/Foxo4 signaling pathway (42). ETV5, belonging to the ETS family of transcription factors, is a key factor in cancer research, recognized for its role in cell cycle regulation and tumor progression. In neuroblastoma, ETV5 drives tumor aggressiveness through transcriptional regulation mediated by activated ALK mutations and is influenced by the MAPK signaling pathway, a mechanism consistent across different cancer types (43). Moreover, ETV5’s oncogenic role in colorectal cancer involves enhancing tumor proliferation and affecting the G1/S transition in the cell cycle, primarily by regulating p21 expression (44). LAMP5, a lysosome-associated membrane protein, plays a crucial role in leukemia and gastric cancer. In leukemia, particularly mixed-lineage leukemia rearrangements (MLL-r), LAMP5 is a direct target of the oncogenic MLL fusion protein, and its reduction significantly inhibits leukemia cell growth, highlighting its potential as a therapeutic target (45). In gastric cancer, the upregulation of LAMP5 in metastatic tissues is associated with enhanced cell proliferation, invasion, migration, and alterations in apoptosis and the cell cycle, indicating its significant role in metastasis formation and potential as a drug development target (46).

In summary, our systematic analysis of dendritic cell heterogeneity in ESCC has identified that tDCs and pDCs can significantly impact the prognosis of ESCC patients. Utilizing marker genes from these two cell groups, we have developed a robust prognostic model that can accurately predict the prognosis and immunotherapeutic efficacy in ESCC. This model could bring new insights into the treatment of ESCC patients. However, we must acknowledge certain limitations. Firstly, as our study relies on existing public data, we lack comprehensive experimental validation of the key genes in our model. This might limit our understanding of the roles these genes play in ESCC. In light of these limitations, our future research will focus on more extensive cellular and animal experiments. These studies will enable us to more directly validate the roles these genes play in the development of ESCC, particularly regarding the functions of dendritic cells. Through these experiments, we hope to provide stronger evidence to support our findings and further deepen our understanding of the heterogeneity of dendritic cells in ESCC and their role in TME.

## Data availability statement

The datasets presented in this study can be found in online repositories. The names of the repository/repositories and accession number(s) can be found in the article/Supplementary Material.

## Ethics statement

The studies involving humans were approved by Research Ethics Committee of the Second Hospital of Hebei Medical University the Ethics Committee of the Tianjin Chest Hospital. The studies were conducted in accordance with the local legislation and institutional requirements. The participants provided their written informed consent to participate in this study.

## Author contributions

MS: Conceptualization, Data curation, Formal analysis, Writing – original draft, Writing – review & editing, Investigation, Methodology, Resources, Software, Supervision, Validation, Visualization. HZ: Formal analysis, Methodology, Project administration, Resources, Software, Validation, Visualization, Writing – original draft, Writing – review & editing. LM: Investigation, Methodology, Software, Writing – original draft, Writing – review & editing. XW: Project administration, Software, Supervision, Writing – original draft. DS: Funding acquisition, Supervision, Writing – review & editing. ZF: Conceptualization, Supervision, Validation, Writing – review & editing, Funding acquisition.
