# Supplementary material for: Innovative prognostic modeling in ESCC: leveraging scRNA-seq and bulk-RNA for dendritic cell heterogeneity analysis
Source: Front Immunol. 2024 Mar 6;15:1352454. doi: 10.3389/fimmu.2024.1352454 (PMC10956130; doi:10.3389/fimmu.2024.1352454)
Supplement: Supplementary file 1 [file DataSheet_1.docx]

Supplementary Material

# Supplementary Data

|  |  | Sequence (5'->3') |
| --- | --- | --- |
| CCDC50 | Forward primer | ATGAAGATGGAGGAATGAAGCCA |
|  | Reverse primer | ATGAAGATGGAGGAATGAAGCCA |
| CR2 | Forward primer | ATATCTGGCAAGGAAGCCCC |
|  | Reverse primer | GAGGTGACTGGCAGCCTTTT |
| ETV5 | Forward primer | CTCCAGACATCATTCCCGGC |
|  | Reverse primer | GGGCATGAAGCACCAGGTTA |
| SDS | Forward primer | TCAGAAGACTGTCCACCCGT |
|  | Reverse primer | CTCCAGACATCATTCCCGGC |
| LAMP5 | Forward primer | TTTGTGGTGCGGGAAAATGG |
|  | Reverse primer | CATGTTGTGGCTTTCATCTACG |
| NEURL3 | Forward primer | GTAACGTGATCTCTGGCCCAT |
|  | Reverse primer | CCTTGGCGTTGGCCTCG |

**Supplement Table1：Primer sequence of the model gene**

## 2 Supplementary Figures


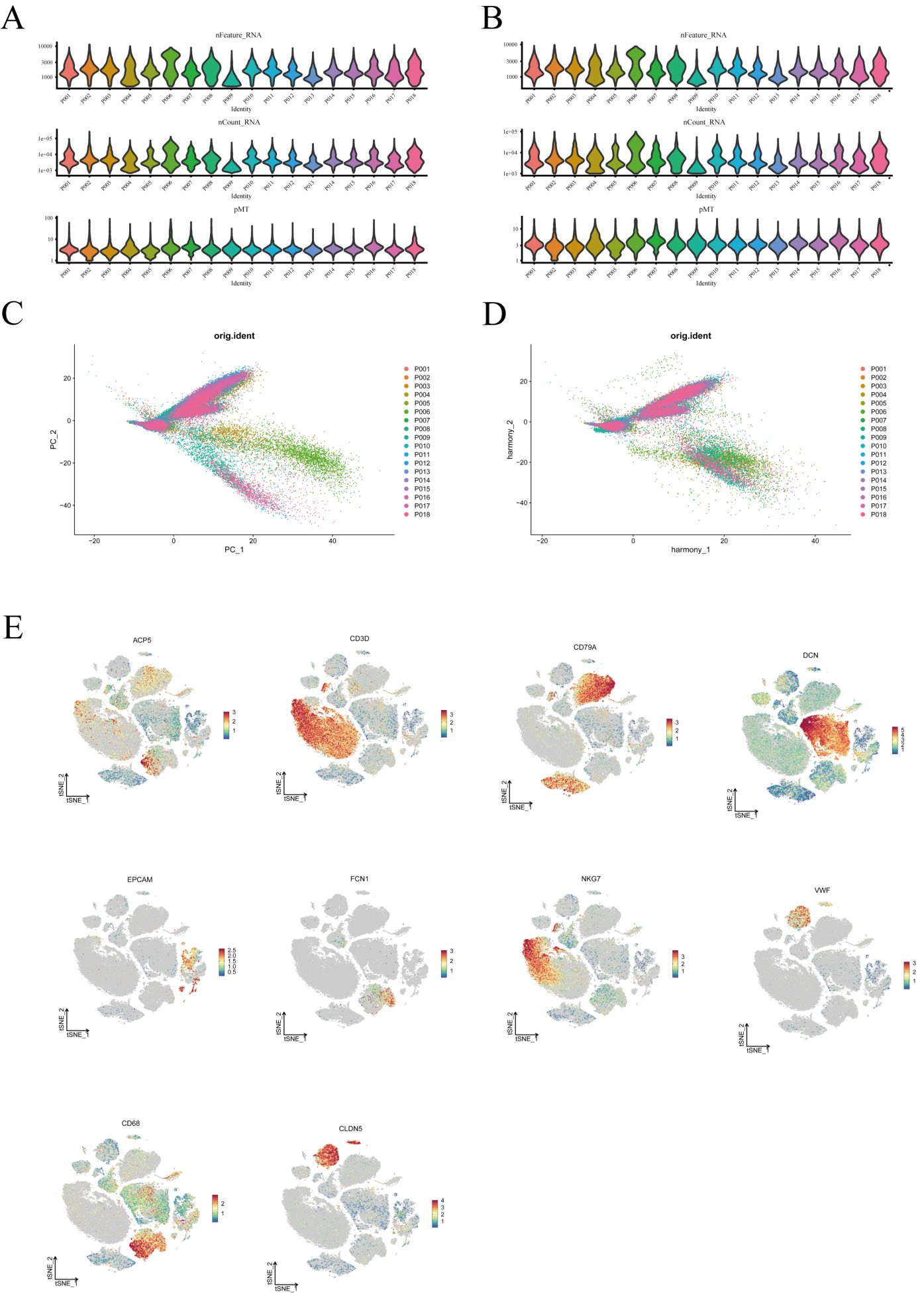


**Supplemental Figure 1: Quality Control and Marker Gene Expression Analysis of Single-Cell RNA Sequencing Data.** (A) Distribution of nFeature (number of genes), nCount (total reads), and pMT (proportion of mitochondrial genes) in each sample before quality control. (B) Distribution of nFeature, nCount, and pMT in each sample after quality control, demonstrating the improvement in data quality post-cleansing. (C) Sample distribution before batch effect removal, showing potential experimental batch-related biases. (D) Sample distribution after batch effect removal, showing a more uniform distribution of samples, indicating effective correction of batch effects. (E) Display of marker gene expression levels in different cell groups, with varying shades of color representing the expression levels of different genes in each cell group, revealing cell group-specific gene expression patterns.


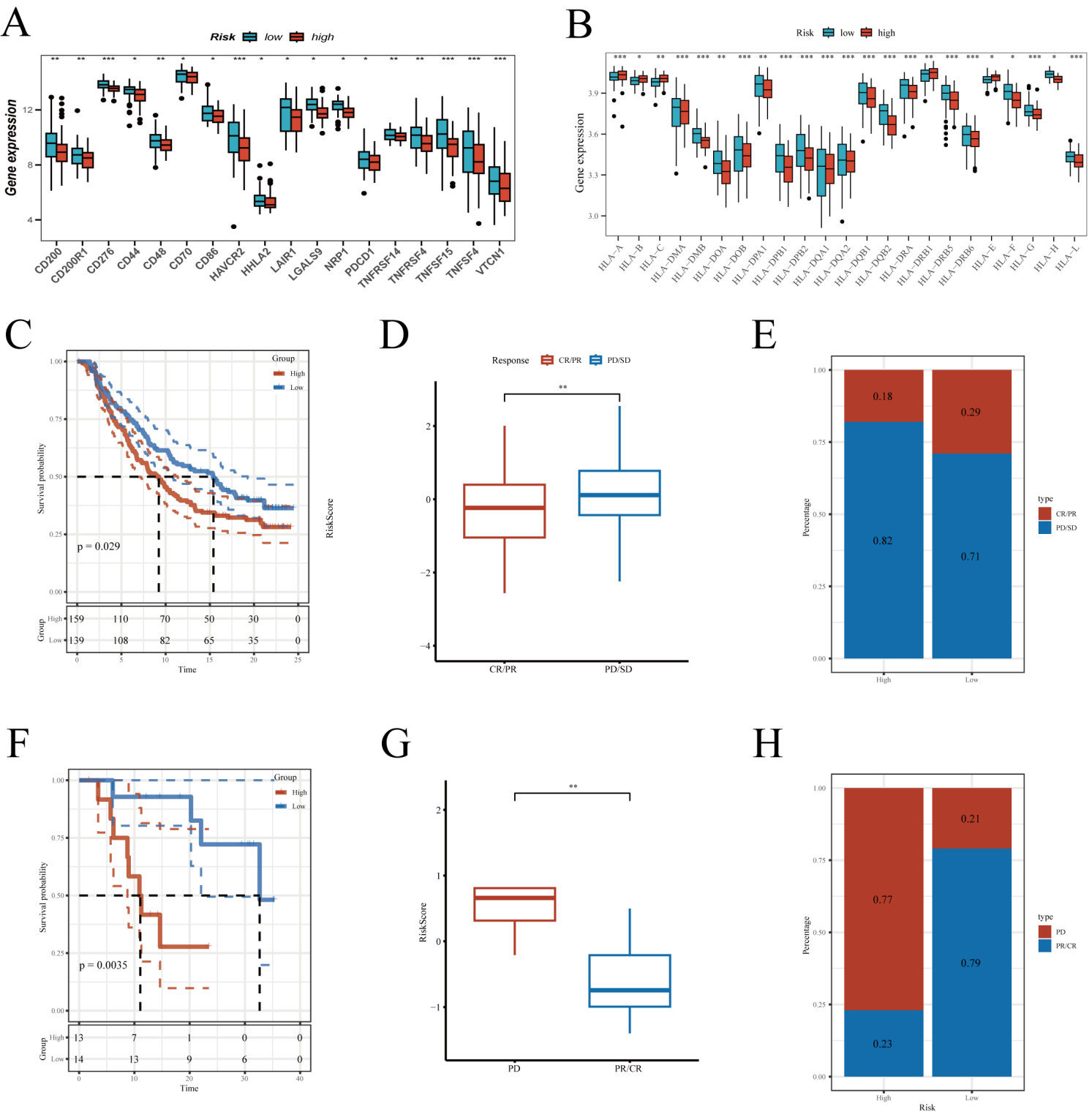


**Supplemental Figure 2: Assessment of Immunotherapy Efficacy.** (A) Differential expression of immune checkpoint-related genes between high and low risk groups. (B) Differential expression of MHC genes between high and low risk groups. (C) Survival curves in the IMvigor210 immunotherapy cohort. (D) Differences in risk scores between different immunotherapy outcomes. (E) Proportion of patients with different immunotherapy efficacies within high and low risk groups. (F) Survival differences between high and low risk groups in the GSE78220 immunotherapy cohort. (G) Differences in risk scores between different immunotherapy outcomes in the GSE78220 cohort. (H) Proportion of patients with different immunotherapy efficacies within high and low risk groups in the GSE78220 cohort.


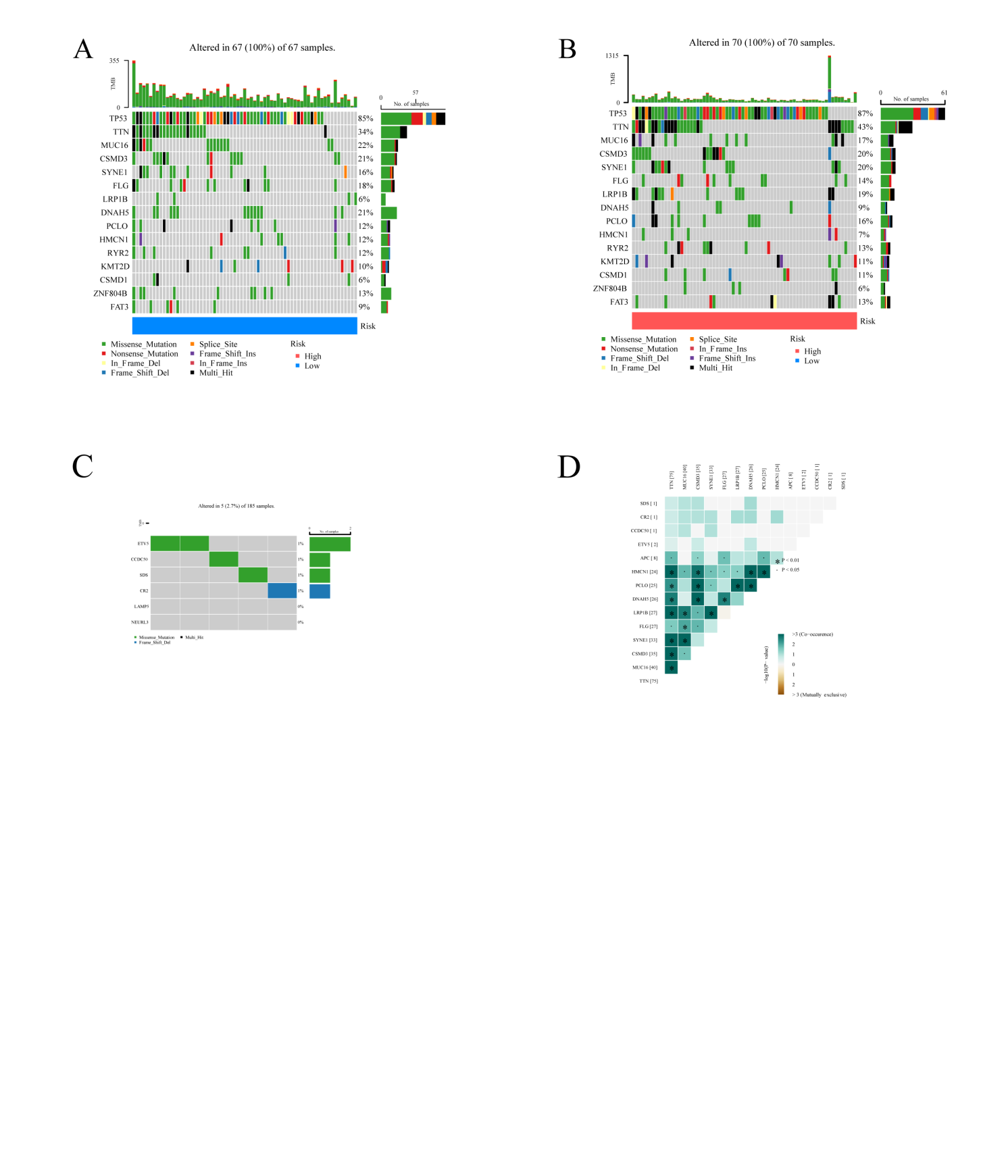


**Supplementary Figure 3: Mutation Information.** (A) Mutation landscape in patients of the low-risk group. (B) Mutation landscape in patients of the high-risk group. (C) Mutation status of Hub genes. (D) Co-mutation information of Hub genes.
